# Comparative larval myogenesis and adult myoanatomy of the rhynchonelliform (articulate) brachiopods *Argyrotheca cordata*, *A. cistellula*, and *Terebratalia transversa*

**DOI:** 10.1186/1742-9994-6-3

**Published:** 2009-02-03

**Authors:** Andreas Altenburger, Andreas Wanninger

**Affiliations:** 1 University of Copenhagen, Department of Biology, Research Group for Comparative Zoology, Universitetsparken 15, DK-2100 Copenhagen Ø, Denmark

## Abstract

**Background:**

Despite significant methodological progress, Brachiopoda remains one of the lophotrochozoan phyla for which no recent ontogenetic data employing modern methodologies such as fluorescence labelling and confocal microscopy are available. This is particularly astonishing given the ongoing controversy concerning its phylogenetic position. In order to contribute new morphogenetic data for phylogenetic and evolutionary inferences, we describe herein the ontogeny and myoanatomy of larvae and adults of the rhynchonelliform brachiopods *Argyrotheca cordata, A. cistellula*, and *Terebratalia transversa *using fluorescence F-actin labelling combined with confocal laserscanning microscopy.

**Results:**

Fully grown larvae of *A. cordata *and *T. transversa *consist of three distinct body regions, namely an apical lobe, a mantle lobe with four bundles of setae, and a pedicle lobe. Myogenesis is very similar in these two species. The first anlagen of the musculature develop in the pedicle lobe, followed by setae muscles and the mantle lobe musculature. Late-stage larvae show a network of strong pedicle muscles, central mantle muscles, longitudinal muscles running from the mantle to the pedicle lobe, setae pouch muscles, setae muscles, a U-shaped muscle, serial mantle muscles, and apical longitudinal as well as apical transversal muscles. Fully developed *A. cistellula *larvae differ from the former species in that they have only two visible body lobes and lack setae. Nevertheless, we found corresponding muscle systems to all muscles present in the former two species, except for the musculature associated with the setae, in larvae of *A. cistellula*. With our survey of the adult myoanatomy of *A. cordata *and *A. cistellula *and the juvenile muscular architecture of *T. transversa *we confirm the presence of adductors, diductors, dorsal and ventral pedicle adjustors, mantle margin muscles, a distinct musculature of the intestine, and striated muscle fibres in the tentacles for all three species.

**Conclusion:**

Our data indicate that larvae of rhynchonelliform brachiopods share a common muscular bodyplan and are thus derived from a common ancestral larval type. Comparison of the muscular phenotype of rhynchonelliform larvae to that of the other two lophophorate phyla, Phoronida and Ectoprocta, does not indicate homology of individual larval muscles. This may be due to an early evolutionary split of the ontogenetic pathways of Brachiopoda, Phoronida, and Ectoprocta that gave rise to the morphological diversity of these phyla.

## Background

Brachiopoda is a small lophophorate phylum with a prominent fossil record since the Lower Cambrium [1]. More than 12.000 fossil and approximately 380 recent species are known to date [2,3]. The phylum is commonly divided into three taxa, the articulate Rhynchonelliformea and the two inarticulate clades Craniiformea and Linguliformea [4], and has traditionally been grouped together with Phoronida and Ectoprocta into the superphylum Lophophorata. However, this classification has recently been challenged by paleontological and molecular datasets. While some analyses employing morphological data assign Brachiopoda to Deuterostomia [e.g., [5,6]], recent molecular data either propose sistergroup relationships to various spiralian phyla including Mollusca, Annelida, and Nemertea [7-11], or support the notion that Phoronida are an ingroup of Brachiopoda [12,13].

Apart from some mainly gross morphological studies [14-21], detailed data using modern techniques such as fluorescence labelling and confocal laserscanning microscopy are not yet available. This is especially true with respect to the development of the musculature, despite the fact that myo-anatomical features may provide useful characters for reconstructing phylogenetic relationships [22,23]. Recently, some data on larval muscle development for the proposed brachiopod sister groups Phoronida and Ectoprocta have become available [24-28]. Accordingly, larval myogenesis in Brachiopoda constitutes an important gap of knowledge in comparative developmental studies on Lophophorata. With the first thorough, comparative account of brachiopod larval myogenesis provided herein for the rhynchonelliform species *Argyrotheca cordata *(Risso, 1826), *Argyrotheca cistellula *(Searles-Wood, 1841), and *Terebratalia transversa *(Sowerby, 1846), we aim at stimulating the discussion concerning lophophorate bodyplan evolution, phylogeny, and development. Furthermore, we contribute to questions concerning the muscular ground pattern of rhynchonelliform brachiopod larvae. We supplement our ontogenetic data with a detailed description of the adult muscle systems of all three species.

## Results

### Embryonic and larval development of *Argyrotheca cordata*

**Figure 1:**
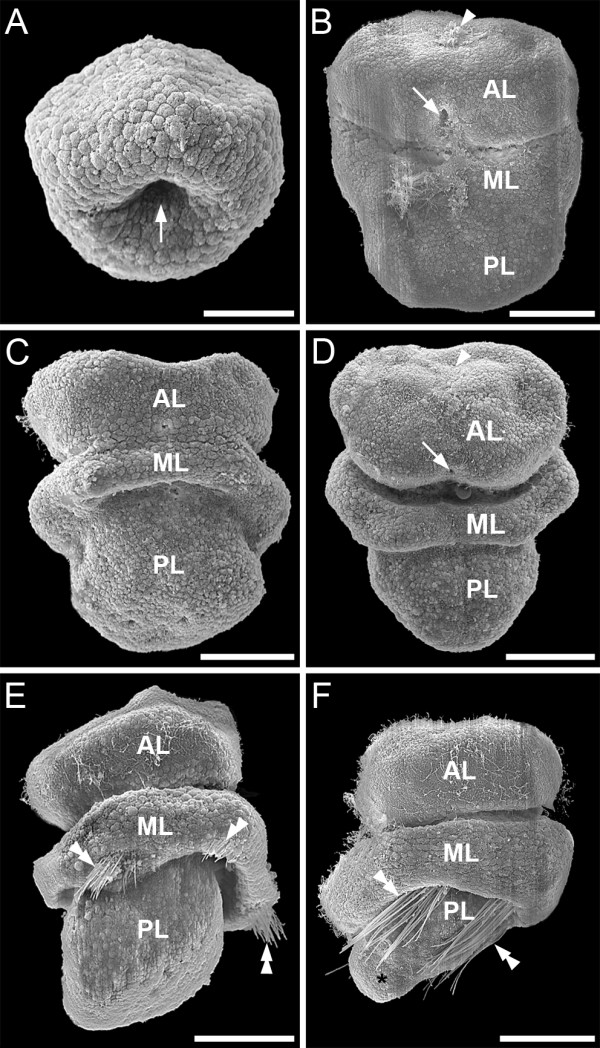
**Scanning electron micrographs of the embryonic and larval development of *Argyrotheca cordata***. Anterior faces upward and scale bars equal 50 μm. (A) Early gastrula with blastopore (arrow). (B) Ventral view of an embryo at the onset of differentiation of the three-lobed larval bodyplan comprising apical lobe (AL), mantle lobe (ML), and pedicle lobe (PL). The arrowhead points to the region of the larval apical ciliary tuft. The arrow points to the larval mouth which corresponds to the blastopore. (C) Dorsal view of a larva with distinct anlagen of the three body lobes. (D) Ventral view of a specimen of the same ontogenetic stage as the one in C with reduced larval apical ciliary tuft (arrowhead) and with the almost closed blastopore (arrow). (E) Three-lobed larva at the onset of setae formation (double arrowheads), dorso-lateral view. (F) Lateral view of a fully differentiated larva showing two of the four pairs of larval setae (double arrowheads) and a distinct primordial hump (asterisk).

Embryos and larvae of *Argyrotheca cordata *are brooded by the mother animal and are released as late-stage larvae competent to undergo metamorphosis. Accordingly, larval development is entirely lecithotrophic. After cleavage and gastrulation (Fig. 1A), a three-lobed larva is established, which comprises an anterior apical lobe, a mantle lobe in the mid-body region, and a posterior pedicle lobe (Fig. 1B–F). In very early three-lobed stages, the blastopore is visible at the base of the apical lobe (Fig. 1B). This larval mouth closes during subsequent larval development (Fig. 1D).

The apical lobe is ciliated and bears, in early three lobed stages, an apical tuft which is lost in later stages (Fig. 1B, D). When the three lobes are fully established, four bundles of larval setae are formed at the posterior margin of the mantle lobe (Fig. 1E). Finally, in larvae competent to undergo metamorphosis, the anlage of the pedicle becomes visible as a distinct primordial hump at the posterior pole of the pedicle lobe (Fig. 1F).

### Myogenesis and adult myoanatomy of *Argyrotheca cordata*

**Figure 2:**
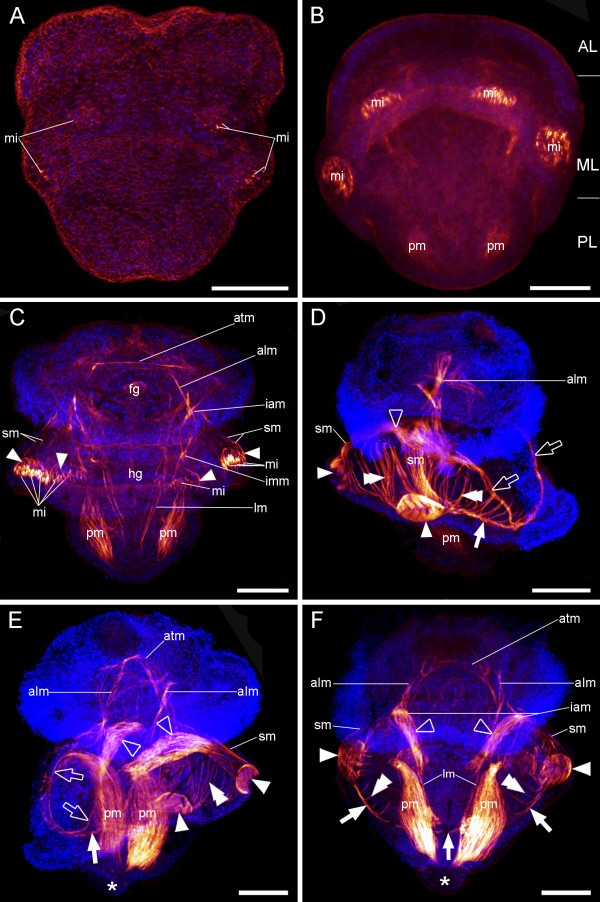
**Myogenesis in *Argyrotheca cordata***. CLSM maximum projection micrographs, anterior faces upward. F-actin is labelled in red and cell nuclei are labelled in blue to indicate the outline of the specimens. Scale bars equal 50 μm. (A) Early larva in dorsal view with the first F-actin signals from microvilli (mi) within the setal canals. (B) Early three-lobed larval stage, postero-dorsal view, showing apical lobe (AL), mantle lobe (ML), pedicle lobe (PL), first rudiments of the pedicle musculature (pm), and microvilli (mi) in the setae pouches. (C) Larval stage with fully differentiated lobes and short setae in ventral view (corresponding to the larval stage shown in Fig. 1E). Visible are the apical transversal muscle (atm), the apical longitudinal muscles (alm), the interconnecting apical muscles (iam), the interconnecting mantle muscles (imm), the longitudinal muscles (lm), the foregut rudiment (fg), the hindgut rudiment (hg), the pedicle muscles (pm), microvilli (mi), the setae pouch musculature (arrowheads), and the setae muscles (sm). (D) Lateral right view of a fully developed three-lobed larva with the U-shaped muscle (empty arrows) on the ventral side. At this stage, the setae pouches are interconnected by a circular mantle muscle (arrow). New at this stage are the central mantle muscles (empty arrowhead). Further indicated are the setae pouch musculature (arrowheads), the setae muscles (sm), the serial mantle muscles (double arrowheads), the pedicle musculature (pm), and the apical longitudinal muscles (alm). (E) Same stage as in D, ventro-lateral view. The U-shaped muscle (empty arrows) is directly connected to the pedicle muscles (pm). In addition, the apical transversal muscle (atm), the apical longitudinal muscles (alm), the serial mantle muscles (double arrowhead), the central mantle muscles (empty arrowheads), the setae pouch muscles (arrowheads), the setae muscles (sm), the circular mantle muscle (arrow), and the primordial hump (asterisk) are indicated. (F) Fully grown larva in ventral view with circular mantle muscle (arrows), serial mantle muscles (double arrowheads), setae pouch muscles (arrowheads), setae muscles (sm), pedicle muscles (pm), longitudinal muscles (lm), apical longitudinal muscles (alm), apical transversal muscle (atm), interconnecting apical muscles (iam), primordial hump (asterisk), and central mantle muscles (empty arrowheads).

The larvae investigated were about 230–270 μm long and 210–240 μm wide. The first F-actin-positive signal is visible as distinct spots in the area that later forms the mantle lobe (Fig. 2A). These distinct spots are F-actin-positive microvilli which are situated in the lower part of the setal sacs where the setae are formed [cf. [29]]. The strong fluorescence signal of the microvilli disappears once setae formation is completed, due to the increasing predominance of the larval musculature (Fig. 2D–F).

The pedicle muscles start to form in three-lobed larvae that still lack setae (Fig. 2B). In older larvae with short setae (corresponding to the stage shown in Fig. 1E), setae muscles start to develop. These run from the setal pouches in anterior direction and connect to the apical longitudinal muscles at the border between apical and mantle lobe (lateral setae muscles) or to the central mantle muscles (dorsal setae muscles), respectively (Fig. 2C). The apical longitudinal muscles extend laterally within the apical lobe and terminate anteriorly at an apical transversal muscle (Fig. 2C). At this stage, longitudinal muscles are also found within the pedicle lobe. From there, they run into the mantle lobe, where they connect to longitudinal muscles which originate at the muscle interconnection point at the border between apical and mantle lobe. The larval gut rudiment is visible as a tube in the centre of the larvae (Fig. 2C).

In fully developed larvae, setae pouch muscles are established and interconnected by a circular mantle muscle (Fig. 2D). From this circular mantle muscle emerge serial mantle muscles, which are dorsolaterally closed by the central mantle muscles. The central mantle muscles are connected to the dorsal setae muscles and to the apical longitudinal muscles at the border of the apical and the mantle lobe (Fig. 2E–F). Anteroventrally, the serial mantle muscles are enclosed by a U-shaped muscle which extends ventrally from the pedicle muscles towards the circular mantle muscle (Fig. 2D–F; see also additional file 1). The primordial hump is devoid of any musculature (Fig. 2E–F).

**Figure 3:**
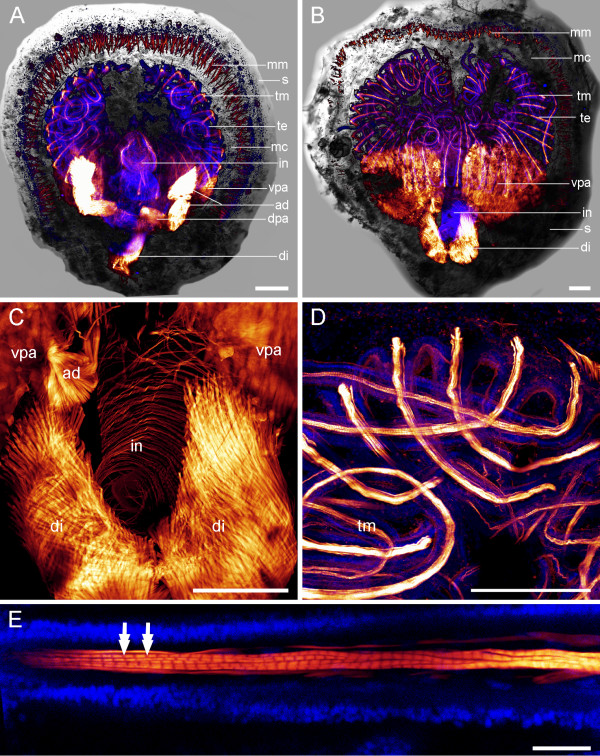
**Adult myoanatomy of *Argyrotheca cordata***. F-actin is labelled in red and cell nuclei are labelled in blue. Scale bars equal 100 μm in all aspects except in E, where it equals 25 μm. (A) Overlay of CLSM maximum projection micrograph and light micrograph, anterior faces upward, dorsal view. Indicated are the tentacle muscles (tm), the mantle margin muscles (mm), the tentacles of the lophophore (te), the mantle cavity (mc), the intestine (in), the shell (s), the adductors (ad), the ventral pedicle adjustors (vpa), which extend from the ventral valve into the pedicle, the dorsal pedicle adjustors (dpa), which extend from the dorsal valve into the pedicle, and the diductors (di). One diductor is lacking as a result of the removal of the animal from the substrate. (B) Overlay of a CLSM maximum projection micrograph and a light micrograph, anterior faces upward, ventral view. Indicated are the same structures as in A. (C) Enlarged view of the ring musculature lining the intestine (in). In addition, one adductor (ad), the diductors (di), and the ventral pedicle adjustors (vpa) are visible. (D) Enlarged view of the tentacles of the lophophore and the corresponding tentacle musculature (tm). (E) Detail of a tentacle muscle fibre showing typical striation pattern (double arrows).

Adult *A. cordata *studied were 0.8–1.3 mm wide and 0.9–1.4 mm long. We can confirm four pairs of muscles which have been described previously [30]. These are one pair of adductors and one pair of diductors, which attach to both the dorsal and to the ventral valve. In addition, there are two pairs of pedicle adjustors, one of which being attached to the ventral valve and the pedicle, and one being attached to the dorsal valve and the pedicle (Fig. 3A–B). In addition, we found a distinct musculature in the tentacles of the lophophore and in the digestive system. Each tentacle contains several bands of striated muscle fibers (Fig. 3D–E), while the stomach and intestine are each lined by numerous delicate ring muscles (Fig. 3C). Moreover, minute muscles are distributed along the dorsal and ventral mantle margin, which probably function as mantle retractor muscles. These mantle retractors are abundant and are oriented perpendicularly to the mantle margin that lines the shell (Fig. 3A–B).

### Myogenesis and adult myoanatomy of *Argyrotheca cistellula*

**Figure 4:**
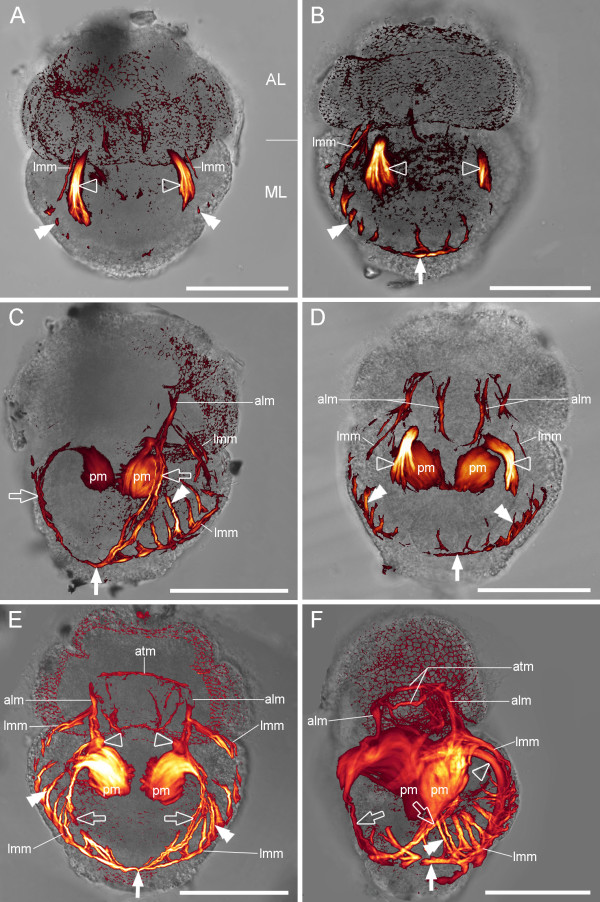
**Myogenesis in *Argyrotheca cistellula***. Overlay of CLSM maximum projection micrograph and light micrograph, anterior faces upward. Scale bars equal 50 μm. Note that only two lobes are visible: the apical lobe (AL) and the mantle lobe (ML), which encloses the pedicle lobe. (A) Early larva in dorsal view with the dorsal mantle muscles (empty arrowheads), the early lateral mantle muscles (lmm), and early rudiments of the serial mantle muscles (double arrowheads). (B) Dorsal view of a later larval stage with the lateral mantle muscle strand (lmm), rudiments of the posterior muscle ring (arrow), dorsal mantle muscles (empty arrowheads), and the serial mantle muscles (double arrowhead). (C) Later larva in ventro-lateral left view with pedicle muscles (pm) that are connected to the ventral mantle muscles (empty arrows). The serial mantle muscles (double arrowhead) are connected to the lateral mantle muscles (lmm), the apical longitudinal muscles (alm) start to develop, and the early posterior muscle ring is visible (arrow). (D) Same stage as in C, dorsal view. The pedicle muscles (pm) are prominent and connect to the dorsal mantle muscles (empty arrowheads). In addition, the lateral mantle muscles (lmm), the serial mantle muscles (double arrowheads), a part of the posterior muscle ring (arrow), and the apical longitudinal muscles (alm) are visible. (E) Fully developed larva, ventral view. The apical transversal (atm) and the apical longitudinal muscles (alm) are fully developed and connect to the pedicle muscles (pm). The connection between pedicle muscles and dorsal mantle muscles (empty arrowheads) is visible in the anterior region of the pedicle muscles. Further indicated are the ventral mantle muscles (empty arrows), the serial mantle muscles (double arrowheads), the lateral mantle muscles (lmm), and the area of the posterior muscle ring (arrow). (F) Same larval stage as in E, ventro-lateral left view. The pedicle muscles (pm) are the most prominent muscles in the centre of the mantle lobe. They are connected to the apical longitudinal muscles (alm), which terminate at the apical transversal muscle (atm), which in turn forms a muscle ring in the apical lobe. The ventral mantle muscles (empty arrows) and dorsal mantle muscles (empty arrowhead) are also connected to the pedicle muscles. The serial mantle muscles (double arrowhead) extend dorsally and ventrally from the lateral mantle muscles (lmm). The latter terminate at the posterior muscle ring (arrow).

Similar to *Argyrotheca cordata*, larvae of *A. cistellula *are lecithotrophic and are brooded by the mother animal. *A. cistellula *larvae lack setae and the mantle lobe encloses the pedicle lobe during development. Thus, the fully developed larvae have only two visible lobes, namely the apical and the mantle lobe. The investigated larvae were around 117–139 μm long and 78–104 μm wide. The first muscles appear in larvae with all lobes fully differentiated. These are two dorsal mantle muscles which extend dorsally from anterior to posterior in the mantle lobe (Fig. 4A). Parallel and further lateral to these dorsal mantle muscles run the early lateral mantle muscles, and the first rudiments of the serial mantle muscles arise at this stage in the mantle lobe. These develop subsequently into a network of muscles that extends dorsally and ventrally from the two lateral mantle muscles (Fig. 4A–F). These lateral mantle muscles connect to the apical longitudinal muscles at the anterior pole and to the posterior muscle ring at the posterior pole of the larvae (Fig 4B–F). During subsequent development, the ventral mantle muscles and the pedicle muscles emerge (Fig. 4C). The pedicle muscles, situated in the centre of the mantle lobe, are the most prominent muscles in fully grown larvae (Fig. 4D). They connect to the apical longitudinal muscles, which in turn are in contact with the apical transversal muscles. The latter form a muscle ring in the apical lobe (Fig. 4E–F). The musculature of fully developed larvae includes the pedicle muscles, which are connected to the apical longitudinal muscles, the ventral mantle muscles, and the dorsal mantle muscles that connect to the pedicle muscles. Furthermore, serial mantle muscles, which extend dorsally and ventrally from the lateral mantle muscles, are present. Ventrally, the serial mantle muscles terminate at the ventral mantle muscles (Fig. 4F).

**Figure 5:**
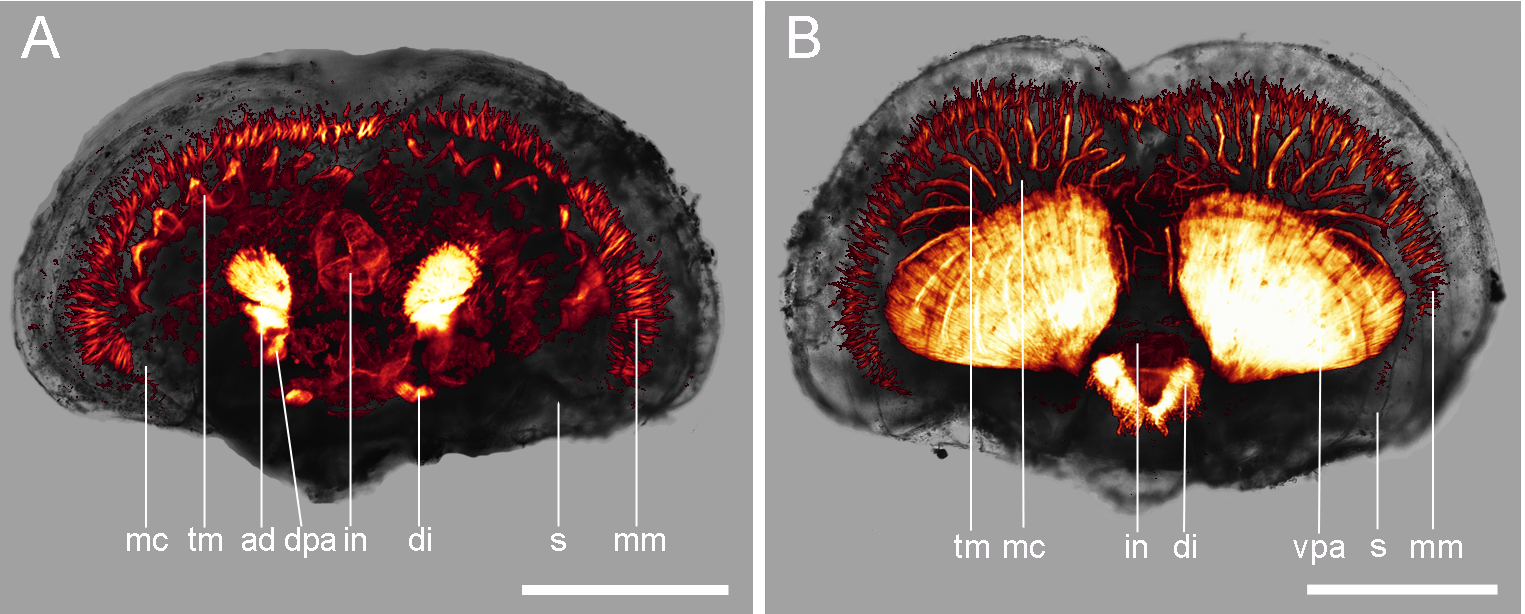
**Adult myoanatomy of *Argyrotheca cistellula***. Overlay of CLSM maximum projection micrograph and light micrograph, anterior faces upward. Scale bars equal 300 μm. (A) Dorsal view. (B) Ventral view. Indicated are the mantle margin muscles (mm), the shell (s), the adductors (ad), the diductors (di), the dorsal pedicle adjustor (dpa), the ventral pedicle adjustor (vpa), the intestine (in), the mantle cavity (mc), and the tentacle muscles (tm).

Similar to the condition found in *Argyrotheca cordata*, four pairs of shell muscles are found in adult *A. cistellula *(Fig. 5). One pair of shell adductors attaches medially to the dorsal and to the ventral valve (Fig. 5). Two pairs of pedicle adjustors extend posterior into the pedicle, whereby one attaches to the dorsal and one to the ventral valve. Finally, one pair of diductors attaches at the posterior end of the ventral valve and runs to the dorsal valve.

Each tentacle of the lophophore contains a number of striated muscle fibres. Mantle margin muscles are arranged perpendicularly to the shell periphery along the edge of the dorsal and the ventral valve (Fig. 5A–B).

### Myogenesis, metamorphosis, and juvenile myoanatomy of *Terebratalia transversa*

**Figure 6:**
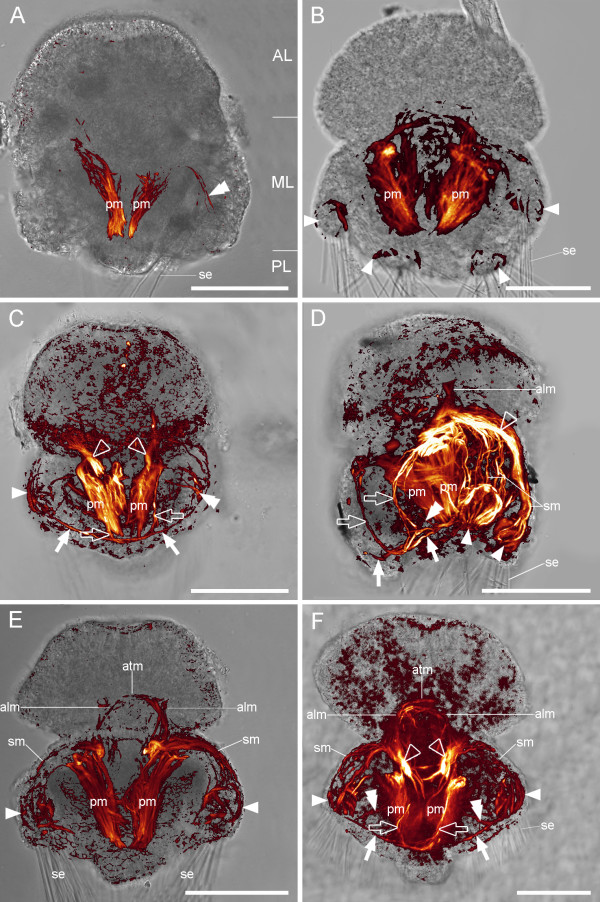
**Myogenesis in *Terebratalia transversa***. Overlay of CLSM maximum projection micrograph and light micrograph, anterior faces upward. Scale bars equal 50 μm. (A) Ventral view of an early three-lobed stage with apical lobe (AL), mantle lobe (ML), and pedicle lobe (PL). Discernable are the pedicle musculature (pm), the first anlagen of the serial mantle muscles (double arrowhead), and the setae (se). (B) Ventral view of a slightly older larva with prominent pedicle musculature (pm), anlagen of the setae pouch musculature (arrowheads), and setae (se). (C) Later larval stage, ventral view with pedicle musculature (pm), setae pouch muscles (arrowhead), serial mantle muscles (double arrowhead), and central mantle muscles (empty arrowheads), which are extensions of the dorsal setae muscles. The serial mantle muscles are posteriorly connected to the circular mantle muscle (arrows) and antero-ventrally connected to the U-shaped muscle (empty arrows), which extends from the pedicle muscles to the circular mantle muscle. (D) Lateral view of a later larva with the muscle systems described in C. In addition, the first anlagen of the apical longitudinal musculature (alm), the setae muscles (sm), and the setae (se) are visible. (E) Same stage as in D with prominent pedicle muscles (pm) that are connected to the apical longitudinal muscles (alm). The latter connect to the apical transversal muscle (atm). In addition, the setae pouch muscles (arrowheads), the setae muscles (sm), and the setae (se) are indicated. (F) Fully developed larva, ventral view, with central mantle muscles (empty arrowheads), pedicle muscles (pm), circular mantle muscle (arrows), U-shaped muscle (empty arrows), serial mantle muscles (double arrowheads), setae pouch musculature (arrowheads), setae muscles (sm), apical longitudinal muscles (alm), apical transversal muscle (atm), and setae (se).

Larvae of *Terebratalia transversa *are lecithotrophic and develop for approximately four days at 11°C in the water column until they are competent to undergo metamorphosis. The investigated larvae were three-lobed, 120–178 μm long and 94–141 μm wide, whereby the pedicle lobe was partly overgrown by the mantle lobe. The first developing muscles are the pedicle muscles and early rudiments of the serial mantle muscles (Fig. 6A). Thereafter, the musculature of the four setae pouches forms (Fig. 6B). In later stages, the setae pouch muscles interconnect with the circular mantle muscle (Fig. 6C). A U-shaped muscle extends on the ventral side of the larvae from the pedicle muscles towards the circular mantle muscle. The serial mantle muscles and the setae muscles span between the circular mantle muscle and the U-shaped muscle strand. The latter run from the setae pouches to the central mantle muscles (Fig. 6D). The central mantle muscles extend from the dorsal setae muscles, which run from the dorsal setae pouches towards the apical lobe. They connect to the apical longitudinal muscles at the border of the apical and the mantle lobe (Fig. 6D). Subsequently, the apical musculature develops, which consists of an apical transversal muscle and two lateral apical longitudinal muscles that are connected to the serial mantle muscles (Fig. 6E). In late three-lobed larvae, the pedicle muscles are, together with the central mantle muscles, the most prominent muscular structures. The central mantle muscles connect to the serial mantle muscles, the setae pouch muscles, the setae muscles, and the apical musculature (Fig. 6F).

**Figure 7:**
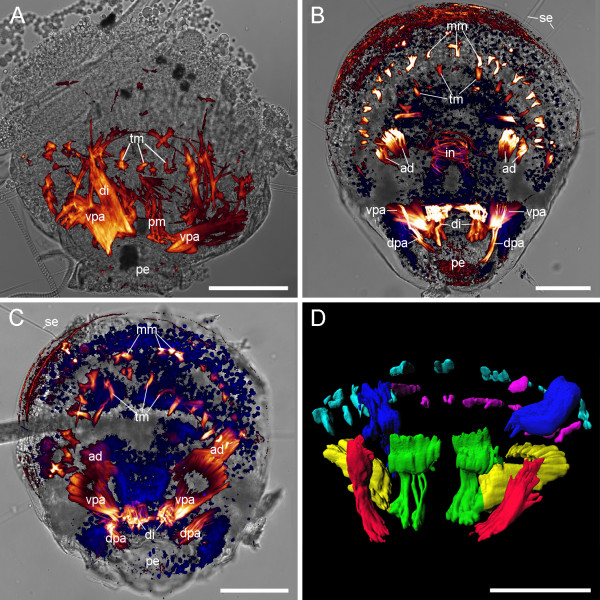
**Metamorphosis and adult myoanatomy of *Terebratalia transversa***. (A-C) Overlay of CLSM maximum projection micrograph and light micrograph, anterior faces upward. F-actin is labelled in red and cell nuclei are labelled in blue. Scale bars equal 50 μm. (A) Larva during metamorphosis. A mosaic of larval and juvenile features are present including the pedicle (pe), the larval pedicle muscles (pm), the first rudiments of the juvenile tentacle musculature (tm), one diductor (di), and the ventral pedicle adjustors (vpa). (B) Juvenile 5 days after metamorphosis, dorsal view with the remaining larval setae (se), the mantle margin muscles (mm), the tentacle muscles (tm), the adductors (ad), the musculature of the intestine (in), the diductors (di), the ventral pedicle adjustors (vpa), the dorsal pedicle adjustors (dpa), and the pedicle (pe). (C) Juvenile 5 days after metamorphosis, ventral view with the remaining larval setae (se), rudiments of the mantle margin muscles (mm), rudiments of the tentacle muscles (tm), the adductors (ad), the ventral pedicle adjustors (vpa), the diductors (di), the dorsal pedicle adjustors (dpa), and the pedicle (pe). (D) Reconstruction of the 3D arrangement of the juvenile musculature based on the CLSM dataset used in C showing the dorsal pedicle adjustors (red), the adductors (dark blue), the mantle margin muscles (light blue), and the tentacle muscles (magenta). The ventral pedicle adjustors (yellow) are ventrally connected to the diductors (green).

During metamorphosis, parts of the larval musculature appear to get resorbed and juvenile muscles develop (Fig. 7A). We were, however, unable to clarify whether or not certain components of the larval musculature are incorporated into the juvenile muscular bodyplan.

The juvenile musculature comprises early rudiments of the tentacle muscles, early rudiments of the mantle margin musculature, the musculature of the intestine, adductors, ventral pedicle adjustors which are connected to the diductors, and dorsal pedicle adjustors (Fig. 7B–D).

## Discussion

### Comparison of larval and adult rhynchonelliform myoanatomy

The gross morphology of *Argyrotheca cistellula *differs considerably from that of *A. cordata *and *Terebratalia transversa *in that the pedicle lobe gets enclosed by the mantle lobe during development [19]. Thus, *A. cistellula *appears two-lobed and lacks setae, while the other two species express three distinct body lobes and setae. Despite these differences, myogenesis follows a similar pattern in all three species (Table 1). When fully developed, prominent pedicle muscles, apical longitudinal as well as apical transversal muscles, and serial mantle muscles are present in all three species. In addition, *A. cordata *and *T. transversa *show a circular mantle muscle which we consider homologous to the posterior muscle ring in *A. cistellula*. This homology is based on the similar position of this muscle in the mantle lobe and the fact that the U-shaped muscle of *A. cordata *and *T. transversa *and the ventral mantle muscles of *A. cistellula *all insert at this muscle. The central mantle muscles of *A. cordata *and *T. transversa *are in our opinion homologous to the dorsal mantle muscles of *A. cistellula *due to the similar position of these muscles and their connection to the apical and the serial mantle muscles in all three species. The U-shaped muscle of *A. cordata *and *T. transversa *corresponds to the ventral mantle muscles in *A. cistellula *due to their similar position and the fact that these muscles enclose the serial mantle muscles antero-ventrally.

**Table 1:** Comparative larval myoanatomy of the rhynchonelliform brachiopods *Argyrotheca cordata*, *Terebratalia transversa*, and *A. cistellula*

	***Species***				
			
**Muscle**	***Argyrotheca cordata***	***Terebratalia transversa***	***Argyrotheca cistellula***	**Location**	**Symbol in figures**
apical longitudinal muscles	+	+	+	apical lobe	alm

apical transversal muscle	+	+	+ (apical muscle ring)	apical lobe	atm

central mantle muscles	+	+	+ (dorsal mantle muscles)	mantle lobe	empty arrowheads

circular mantle muscle	+	+	+ (posterior muscle ring)	mantle lobe	arrows

lateral mantle muscle	-	-	+	mantle lobe	lmm

longitudinal muscles	+	+	-	mantle and pedicle lobe	lm

pedicle muscles	+	+	+	pedicle lobe	pm

serial mantle muscles	+	+	+	mantle lobe	double arrowsheads

setae muscles	+	+	-	mantle lobe	sm

setae pouch musculature	+	+	-	mantle lobe	arrowheads

U-shaped muscle	+	+	+ (ventral mantle muscle)	mantle lobe	empty arrows

Despite these similarities, we found distinct differences in the myoanatomy of the three species investigated. As such, the setae pouch muscles, the setae muscles, and the longitudinal muscles, which run from the mantle lobe to the pedicle lobe, are only present in *A. cordata *and *T. transversa*, while the lateral mantle muscles are only present in larvae of *A. cistellula*. These differences between *A. cistellula *on the one hand and *A. cordata *and *T. transversa *on the other correspond to the gross morphological observation that *A. cistellula *lacks setae.

Larval setae in brachiopods have been proposed to function as a defence device and to control buoyancy [31]. The setae of *A. cistellula *larvae have probably been secondarily lost, as these larvae are brooded and may settle shortly after release from the mother animal. However, *A. cordata *larvae have retained their setae despite being brooded, which may hint towards an extended planktonic period of these larvae.

The muscles in the pedicle lobe have been proposed earlier to be of functional use during metamorphosis [32,33]. When larvae settle, a glandular region at the tip of the primordial hump functions as site of attachment to the substrate [34]. Subsequently, the primordial hump forms the first rudiment of the juvenile pedicle. After larval settlement, the mantle lobe is inverted over the apical lobe and eventually forms the juvenile mantle. The apical lobe gets enclosed by the valves and forms the lophophore and all anterior adult structures [32,35]. At the onset of metamorphosis, the U-shaped muscle may, due to its connection to the pedicle muscles and the circular mantle muscle, aid in inverting the mantle lobe. During metamorphosis, the larval pedicle muscles are still present at the time of ventral pedicle adjustor and diductor formation. However, whether the larval pedicle muscles are resorbed or are (partly) incorporated into the juvenile diductor and/or pedal adjustor muscles could not be clarified by the present study.

*Argyrotheca cordata *is the sole species from this study for which data on the larval myoanatomy had previously been available. In the first descriptions from 1873 and 1883, "muscles abdominaux", that run from the pedicle lobe into the mantle lobe, had been identified [14,30]. A different description was given slightly later, when a network of muscles in the fully developed larva was described. The muscles were denoted "Muskel des lateralen Borstenbündels", "Muskel des medialen Borstenbündels", "musculus contractor", "musculus rotator dorsalis", and "musculus abductor" [15]. Our findings confirm the results of the first papers with respect to the pedicle muscles and the setae muscles. However, in our specimens, the pedicle muscles were not directly connected to the setae muscles as depicted in the first descriptions, but were instead connected to the U-shaped muscle.

In adult *Argyrotheca cordata*, four pairs of muscles had been identified previously [30]. The pair of adductor muscles has two insertion sites, one anterior to the other at the dorsal valve, and an additional one at the ventral valve. The pair of diductor muscles inserts at the posterior part of both the ventral and the dorsal valve. One of the two pairs of adjustors inserts at the ventral valve and the pedicle, while the other pair inserts at the dorsal valve and the pedicle [30].

The muscular systems of adult *A. cordata *and *A. cistellula *are similar to each other and comprise one pair of adductors, two pairs of pedicle adjustors and one pair of diductors. The tentacles contain several fibres of striated musculature which have previously been described as "rows of striated fusiform myoepithelial cells" in the lophophore of *T. transversa *[36].

For the juvenile musculature of *Terebratalia transversa *we followed the nomenclature used by Eshleman and Wilkens [37]. The juvenile musculature, five days after metamorphosis, comprises rudiments of the tentacle muscles, rudiments of the mantle margin musculature, one pair of adductors, one pair of diductors, one pair of dorsal, and one pair of ventral pedicle adjustors. The ventral pedicle adjustors are connected to the diductors in the juvenile.

### Comparative myogenesis of Lophophorata

For the Phoronida, data on muscle development are currently available for three species, namely *Phoronis pallida, P. harmeri*, and *P. architecta *[24,26,27]. The larvae of these species are of the actinotroch-type and differ considerably from brachiopod larvae in both their gross anatomy and in their lifestyle, because these phoronid larvae are planktotrophic, while the brachiopod larvae investigated herein are of the typical three-lobed, lecithotrophic type. Accordingly, a considerable part of the larval phoronid musculature is linked to the digestive system (e.g., the oesophageal ring muscles) and to the maintenance of a cylindrical body shape (e.g., a meshwork of circular and longitudinal muscles in the bodywall). In addition, trunk retractor muscles, that originate from the posterior collar ring muscles and insert in the telotrochal region, are present in phoronid larvae [27]. The collar region contains mainly ring muscles and few longitudinal muscles. The subumbrellar and exumbrellar layers of the hood contain circular muscles and a series of longitudinal muscles, which, in the exumbrellar layer, function as hood elevators [27]. Furthermore, the tentacles of phoronid actinotroch larvae contain elevator and depressor muscles which consist of two loops in the elevators and a single loop in the depressors. These tentacle muscles are interconnected by the ring muscle of the collar [27]. We did not identify any muscles in the larvae of the three brachiopod species described herein that could potentially correspond to the actinotroch muscle systems known so far.

The muscular architecture in ectoproct larvae is very diverse, thus following the high plasticity of larval gross morphology and the notion that lecithotrophic larvae might have evolved up to six times within Ectoprocta [38]. To date, the larval muscular systems have been described for *Membranipora membranacea *(cyphonautes larva), *Flustrellidra hispida *(pseudocyphonautes larva), *Celleporaria sherryae *and *Schizoporella floridana *(both coronate larva), *Bowerbankia gracilis *(vesiculariform larva), *Bugula stolonium *and *B. fulva *(both buguliform larva), *Sundanella sibogae, Nolella stipata, Amathia vidovici, Aeverrillia setigera*, and *Alcyonidium gelatinosum *(all ctenostome larva), and *Crisia elongata *(cyclostome larva) [25,28]. Recently, a number of homologies have been proposed for various larval ectoproct muscle systems [25]. These are the coronal ring muscle, which underlies the ciliated, ring-shaped swimming organ of most larval types, the anterior median muscle, which runs anteriorly from ventral to dorsal in most species, lateral muscles, which project laterally in dorso-ventral direction in most larvae, longitudinal muscles along the posterior body axis, and transversal muscles, which are situated transversally in the central body region of *F. hispida*, *M. membranacea*, and *A. gelatinosum*. Besides these proposed homologous muscles, each larval type shows unique muscles in the body wall and/or inside the larval body, reflecting at least partly the functional adaptations to a planktotrophic *versus *a lecithotrophic lifestyle. No muscles corresponding to any of the ectoproct muscle types were found in the brachiopod species investigated in this study (and noticeably no homologous muscles between the lecithotrophic ectoproct and brachiopod larval types could be identified), again demonstrating the high plasticity of lophophorate larval anatomy.

## Conclusion

All rhynchonelliform brachiopod larvae studied to date are three-lobed with four bundles of setae [39], except for the larva of *Argyrotheca cistellula*, which is externally bilobed and lacks setae, and the three-lobed thecideid larvae, which likewise lack setae [40]. Despite these gross morphological differences, myogenesis in the three brachiopod species investigated is very similar. Thus, we propose a larval muscular groundpattern for rhynchonelliform brachiopods comprising apical longitudinal muscles, apical transversal muscles, circular mantle muscles, central mantle muscles, longitudinal muscles, serial mantle muscles, pedicle muscles, setae pouch muscles, setae muscles, and a U-shaped muscle. However, a final statement can only be made once data on the musculature of theceid and rhynchonellid larvae become available.

Comparing this proposed larval muscular groundpattern to the hitherto investigated phoronids, ectoprocts, and spiralian taxa such as polychaetes, molluscs, plathelminths or entoprocts does not reveal any homologies of larval brachiopod muscles and the muscles of other lophotrochozoan larvae, regardless of whether the respective larvae are lecithotrophic or planktotrophic [23,41-47]. From these data we conclude that the ontogenetic pathways of the individual lophophorate phyla have split early in evolution from that of other Lophotrochozoa, which then resulted in the wide morphological diversity of larval and adult lophophorate bodyplans.

## Methods

### Animal collection and fixation

#### *Argyrotheca cordata *and *A. cistellula*

Adults were obtained from encrusting coralline red algae (coralligène), which was collected in the vicinity of the Observatoire Océanologique de Banyuls-sur-mer, France (42°29'27.51" N; 3°08'07.67" E), by SCUBA from 30–40 m depth in July 2002 and June 2007. All developmental stages from unfertilized eggs to fully differentiated larvae were obtained by dissection from the adults. The specimens were relaxed at room temperature in 7.14% MgCl_2_, fixed in 4% paraformaldehyde (PFA) in 0.1 M phosphate buffer (PB) for 2 hours or for 3–5 hours, and subsequently washed thrice with 0.1 M PB for 15 min each. The samples were stored in 0.1 M PB with 0.1% NaN_3 _at 4°C. Material fixed for 2 hours was used for immunocytochemistry (ICC) and material fixed for 3–5 hours was used for scanning electron microscopy (SEM).

#### *Terebratalia transversa*

Adults were collected in the San Juan Archipelago, USA, in the vicinity of the Friday Harbor Laboratories, and were kept in running seawater tables. To obtain larvae, females were dissected and their eggs transferred into beaker glasses with filtered seawater. The seawater was changed several times in order to wash off follicle cells, and the eggs were left overnight for germinal vesicle breakdown. Males were opened and left in filtered seawater overnight. Thereafter, their testes were scraped out, macerated, and diluted with filtered seawater to obtain a sperm suspension. Prior to fertilization, sperm cells were activated by adding three drops of a 1 M Tris buffer solution (Sigma-Aldrich, St. Louis, MO, USA) to approximately 50 ml of sperm suspension. Larvae were maintained in embryo dishes at around 11°C and the filtered seawater was changed twice daily. Free swimming larvae, metamorphic stages, and juveniles five days after metamorphosis were relaxed in 7.14% MgCl_2 _and fixed in 4% PFA in 0.1 M PB for 30 min at room temperature. Larvae were washed thrice for 15 min in 0.1 M PB and stored in 0.1 M PB with 0.1% NaN_3 _at 4°C.

### Scanning electron microscopy

For scanning electron microscopy (SEM), the specimens were postfixed in 1% OsO_4_, dehydrated in a graded acetone series, critical point dried, and sputter coated with gold. Digital images were acquired using a LEO 1430 VP SEM (Zeiss, Jena, Germany).

### F-actin labelling, confocal laserscanning microscopy (CLSM), and 3D reconstruction

Prior to staining, larvae were washed thrice for 15 min in PB and incubated for 1 h in PB containing 0.1% Triton X-100 (Sigma-Aldrich) to permeabilize the tissue. Then, the specimens were incubated in 1:40 diluted Alexa Fluor 488 phalloidin (Invitrogen, Molecular Probes, Eugene, OR, USA) and 3 μg/ml DAPI (Invitrogen) in the permeabilization solution overnight at 4°C. Subsequently, specimens were washed thrice for 15 min in 0.1 M PB and embedded in Fluoromount G (Southern Biotech, Birmingham, AL, USA) on glass slides. The same procedure was used for juveniles and adults, with the addition of a decalcifying step using 0.05 M EGTA (Sigma-Aldrich) at room temperature overnight prior to permeabilization and staining. Negative controls omitting the phalloidin dye were performed on all species in order to avoid potential misinterpretations caused by autofluorescence.

The samples were analysed with a Leica DM RXE 6 TL fluorescence microscope equipped with a TCS SP2 AOBS laserscanning device (Leica Microsystems, Wetzlar, Germany). Animals were scanned at intervals of 0.49 μm or 0.64 μm, respectively, and the resulting image stacks were merged into maximum projection images. Photoshop CS3 (Adobe, San Jose, CA, USA) was used to create overlay images of CLSM and light micrographs and for assembling the figure plates. 3D reconstruction was performed on CLSM datasets using volume rendering algorithms of the graphics software Imaris 5.7.2 (Bitplane, Zurich, Switzerland).

## Competing interests

The authors declare that they have no competing interests.

## Authors' contributions

AA performed research and drafted the manuscript. AW designed and coordinated research, performed the SEM analysis, and contributed significantly to the writing of the manuscript. Both authors read and approved the final version of the manuscript.

## Supplementary Material

Additional file 1 **Larval musculature of *Argyrotheca cordata*.** Movie of a confocal scan through a fully developed larva of *Argyrotheca cordata *to illustrate the three-dimensional arrangement of the larval musculature. Click here for file
